# Supplementary material for: A multi-omics study of circulating phospholipid markers of blood pressure
Source: Sci Rep. 2022 Jan 12;12:574. doi: 10.1038/s41598-021-04446-7 (PMC8755711; doi:10.1038/s41598-021-04446-7)
Supplement: Supplementary file 1 — Supplementary Information. [file 41598_2021_4446_MOESM1_ESM.docx]

Supplementary Material 1

Protocol for high-throughput quantification of distinct LPC species by ESI-MS/MS (for more information see <https://www.lipidomicnet.org/index.php/Lysophosphatidylcholine_-_ESI-MS/MS_-_Liebisch_et_al.>)^1^

| Sample preparation | - Lipid extraction according to Bligh and Dyer^2^ - Internal standards are added prior lipid extraction - To avoid loss of lipids, extraction was performed in glassware - Samples are dissolved in methanol containing 10 mM ammonium acetate/chloroform (3/1 = v/v)  \| Material \| Material used \| Internal Standard(s) \| Internal Standard(s) added \| \| --- \| --- \| --- \| --- \| \| Cultured cells \| 100µg protein \| LPC 13:0, LPC 19:0 \| 50ng each \| \| Human plasma \| 20µl \| LPC 13:0, LPC 19:0 \| 1500ng each \| |
| --- | --- | --- | --- | --- | --- | --- | --- | --- | --- | --- | --- | --- | --- |
| Instrumentation and method | Pump   - Type: binary high pressure gradient (Agilent 1100) - Mode: isocratic flow gradient - Solvent(s): Methanol containing 10 mM ammonium acetate/chloroform (3/1 = v/v) - Flow gradient:  \| Time [min] \| Flow [ml/min] \| % Solvent A \| % Solvent B \| \| --- \| --- \| --- \| --- \| \| 0 \| 0.05 \| 100 \| 0 \| \| 0.1 \| 0.03 \| 100 \| 0 \| \| 1.1 \| 0.2 \| 100 \| 0 \| \| 1.3 \| 0.05 \| 100 \| 0 \|   Autosampler   - Type: CTC Pal - Injection volume: 20µl - Wash solvent: methanol/chloroform = 1/1   Mass spectrometer   - Type: Triple quadrupole (Micromass, Quattro Ultima) - Source temperature: 300°C - Ionization mode: ESI positive - Ionization voltage: 3500 V - Collision gas: Argon - Collision gas pressure: 1.0 10^-3^ Torr - Collision energy: 24 V - MS/MS-mode: precursor ion scan of m/z 184.1 |
| Data analysis and quantification | Data handling   - Combine spectra above half peak height - Smooth combined spectrum (if necessary), centroid combined spectrum, pick peak intensities   Isotope correction   - Using Excel Macros correcting the peak intensities in a sequential algorithm starting from low mass species - Five isotope peaks including the monoisotopic were used - The detailed algorithm is described in the appendix of Liebisch G, et al’s study^3^   Calibration and quantification   - Calibration type: matrix calibration - addition of naturally occurring species - Species used for calibration:  \| Species \| Cultured cells \| Human plasma \| \| --- \| --- \| --- \| \| LPC 16:0 \| 0 - 100 pmol \| 0 - 1000 pmol \| \| LPC 18:0 \| 0 - 100 pmol \| 0 - 1000 pmol \| \| LPC 18:1 \| 0 - 100 pmol \| 0 - 1000 pmol \| |
| Method validation | Precision   - CV within-run: 3 % (major), 12 % (minor), CV total: 12 % (major), 25 % (minor)   Detection limit   - 0.6 - 0.8µM in plasma |

Supplementary Material 2

Protocol for high-throughput quantification of distinct PLPE species by ESI-MS/MS (for more information see <https://www.lipidomicnet.org/index.php/Phosphatidylethanolamine-based_plasmalogens_-_ESI-MS/MS_-_Liebisch_et_al.>)^3,4^

| Sample preparation | - Lipid extraction according to Bligh and Dyer^2^ - Internal standards are added prior lipid extraction - To avoid loss of lipids, extraction was performed in glassware - Samples are dissolved in methanol containing 10 mM ammonium acetate/chloroform (3/1 = v/v)  \| Material \| Material used \| Internal Standard(s) \| Internal Standard(s) added \| \| --- \| --- \| --- \| --- \| \| Cultured cells \| 100µg protein \| PE 28:0, PE 40:0 (diphytanoyl) \| 500ng each \| \| Human plasma \| 20µl \| PE 28:0, PE 40:0 (diphytanoyl) \| 400ng each \| |
| --- | --- | --- | --- | --- | --- | --- | --- | --- | --- | --- | --- | --- | --- |
| Instrumentation and method | Pump   - Type: binary high pressure gradient (Agilent 1100) - Mode: isocratic flow gradient - Solvent(s): Methanol containing 10 mM ammonium acetate/chloroform (3/1 = v/v) - Flow gradient:  \| Time [min] \| Flow [ml/min] \| % Solvent A \| % Solvent B \| \| --- \| --- \| --- \| --- \| \| 0 \| 0.05 \| 100 \| 0 \| \| 0.1 \| 0.03 \| 100 \| 0 \| \| 1.1 \| 0.2 \| 100 \| 0 \| \| 1.3 \| 0.05 \| 100 \| 0 \|   Autosampler   - Type: CTC Pal - Injection volume: 20µl - Wash solvent: methanol/chloroform = 1/1   Mass spectrometer   - Type: Triple quadrupole (Micromass, Quattro Ultima) - Source temperature: 300°C - Ionization mode: ESI positive - Ionization voltage: 3500 V - Collision gas: Argon - Collision gas pressure: 1.0 10^-3^ Torr - Collision energy: 18 V - MS/MS-mode: - PE p16:0/x: neutral loss scan of m/z 364.3 - PE p18:1/x: neutral loss scan of m/z 390.3 - PE p18:0/x: neutral loss scan of m/z 392.3 |
| Data analysis and quantification | Data handling   - Combine spectra above half peak height - Smooth combined spectrum (if necessary), centroid combined spectrum, pick peak intensities   Isotope correction   - Using Excel Macros correcting the peak intensities in a sequential algorithm starting from low mass species - Five isotope peaks including the monoisotopic were used - The detailed algorithm is described in the appendix of Liebisch G, et al’s study^3^   Calibration and quantification   - Calibration type: matrix calibration - addition of naturally occurring species - Species used for calibration:  \| Species \| Cultured cells \| Human plasma \| \| --- \| --- \| --- \| \| PE p16:0/20:4 \| 0 - 500 pmol \| 0 - 250 pmol \| |

Supplementary Material 3

Protocol for high-throughput quantification of distinct PC and SPM species by ESI-MS/MS (for more information see <https://www.lipidomicnet.org/index.php/Phosphatidylcholine_/_Sphingomyelin_-_ESI-MS/MS_-_Liebisch_et_al.>)^3^

| Sample preparation | - Lipid extraction according to Bligh and Dyer^2^ - Internal standards are added prior lipid extraction - To avoid loss of lipids, extraction was performed in glassware - Samples are dissolved in methanol containing 10 mM ammonium acetate/chloroform (3/1 = v/v)  \| Material \| Material used \| Internal Standard(s) \| Internal Standard(s) added \| \| --- \| --- \| --- \| --- \| \| Cultured cells \| 100µg protein \| PC 28:0, PC 44:0 \| 1250ng each \| \| Human plasma \| 20µl \| PC 28:0, PC 44:0 \| 3500ng PC 28:0, 5000ng PC 44:0 \| |
| --- | --- | --- | --- | --- | --- | --- | --- | --- | --- | --- | --- | --- | --- |
| Instrumentation and method | Pump   - Type: binary high pressure gradient (Agilent 1100) - Mode: isocratic flow gradient - Solvent(s): Methanol containing 10 mM ammonium acetate/chloroform (3/1 = v/v) - Flow gradient:  \| Time [min] \| Flow [ml/min] \| % Solvent A \| % Solvent B \| \| --- \| --- \| --- \| --- \| \| 0 \| 0.05 \| 100 \| 0 \| \| 0.1 \| 0.03 \| 100 \| 0 \| \| 1.1 \| 0.2 \| 100 \| 0 \| \| 1.3 \| 0.05 \| 100 \| 0 \|   Autosampler   - Type: CTC Pal - Injection volume: 20µl - Wash solvent: methanol/chloroform = 1/1   Mass spectrometer   - Type: Triple quadrupole (Micromass, Quattro Ultima) - Source temperature: 300°C - Ionization mode: ESI positive - Ionization voltage: 3500 V - Collision gas: Argon - Collision gas pressure: 1.0 10^-3^ Torr - Collision energy: 30 V - MS/MS-mode: precursor ion scan of m/z 184.1 |
| Data analysis and quantification | Data handling   - Combine spectra above half peak height - Smooth combined spectrum (if necessary), centroid combined spectrum, pick peak intensities   Isotope correction   - Using Excel Macros correcting the peak intensities in a sequential algorithm starting from low mass species - Five isotope peaks including the monoisotopic were used - The detailed algorithm is described in the appendix of Liebisch G, et al’s study^3^   Calibration and quantification   - Calibration type: matrix calibration - addition of naturally occurring species - Species used for calibration:  \| Species \| Cultured cells \| Human plasma \| \| --- \| --- \| --- \| \| PC 34:1 \| 0 - 1000 pmol \| 0 - 3000 pmol \| \| PC 36:2 \| 0 - 1000 pmol \| 0 - 3000 pmol \| \| PC 38:4 \| 0 - 1000 pmol \| 0 - 3000 pmol \| \| PC 40:0 \| 0 - 1000 pmol \| 0 - 3000 pmol \| \| PC O 16:0/20:4 \| 0 - 1000 pmol \| 0 - 3000 pmol \| \| SM 16:0 \| 0 - 1000 pmol \| 0 - 3000 pmol \| \| SM 18:1 \| 0 - 1000 pmol \| 0 - 3000 pmol \| \| SM 18:0 \| 0 - 1000 pmol \| 0 - 3000 pmol \| |
| Method validation | Precision   - CV within-run: 4 % (major), 5-10 % (minor); CV total: 10 % (major), 15 % (minor)   Detection limit   - 0.6 µM in plasma |

Supplementary Material 4

Protocol for high-throughput quantification of distinct PE species by ESI-MS/MS (for more information see <https://www.lipidomicnet.org/index.php/Phosphatidylethanolamine_-_ESI-MS/MS_-_Liebisch_et_al.>)^3,5^

| Sample preparation | - Lipid extraction according to Bligh and Dyer^2^ - Internal standards are added prior lipid extraction - To avoid loss of lipids, extraction was performed in glassware - Samples are dissolved in methanol containing 10 mM ammonium acetate/chloroform (3/1 = v/v)  \| Material \| Material used \| Internal Standard(s) \| Internal Standard(s) added \| \| --- \| --- \| --- \| --- \| \| Cultured cells \| 100µg protein \| PE 28:0, PE 40:0 (diphytanoyl) \| 500ng each \| \| Human plasma \| 20µl \| PE 28:0, PE 40:0 (diphytanoyl) \| 400ng each \| |
| --- | --- | --- | --- | --- | --- | --- | --- | --- | --- | --- | --- | --- | --- |
| Instrumentation and method | Pump   - Type: binary high pressure gradient (Agilent 1100) - Mode: isocratic flow gradient - Solvent(s): Methanol containing 10 mM ammonium acetate/chloroform (3/1 = v/v) - Flow gradient:  \| Time [min] \| Flow [ml/min] \| % Solvent A \| % Solvent B \| \| --- \| --- \| --- \| --- \| \| 0 \| 0.05 \| 100 \| 0 \| \| 0.1 \| 0.03 \| 100 \| 0 \| \| 1.1 \| 0.2 \| 100 \| 0 \| \| 1.3 \| 0.05 \| 100 \| 0 \|   Autosampler   - Type: CTC Pal - Injection volume: 20µl - Wash solvent: methanol/chloroform = 1/1   Mass spectrometer   - Type: Triple quadrupole (Micromass, Quattro Ultima) - Source temperature: 300°C - Ionization mode: ESI positive - Ionization voltage: 3500 V - Collision gas: Argon - Collision gas pressure: 1.0 10^-3^ Torr - Collision energy: 20 V - MS/MS-mode: precursor ion scan of m/z 141.0 |
| Data analysis and quantification | Data handling   - Combine spectra above half peak height - Smooth combined spectrum (if necessary), centroid combined spectrum, pick peak intensities   Isotope correction   - Using Excel Macros correcting the peak intensities in a sequential algorithm starting from low mass species - Five isotope peaks including the monoisotopic were used - The detailed algorithm is described in the appendix of Liebisch G, et al’s study^3^   Calibration and quantification   - Calibration type: matrix calibration - addition of naturally occurring species - Species used for calibration:  \| Species \| Cultured cells \| Human plasma \| \| --- \| --- \| --- \| \| PE 34:1 \| 0 - 500 pmol \| 0 – 250 pmol \| \| PE 36:2 \| 0 - 500 pmol \| 0 – 250 pmol \| \| PE 38:4 \| 0 - 500 pmol \| 0 – 250 pmol \| \| PE 40:6 \| 0 - 500 pmol \| 0 – 250 pmol \| |

Supplementary Material 5

Protocol for high-throughput quantification of distinct ceramide (CER) species by ESI-MS/MS (for more information see [https://www.lipidomicnet.org/index.php/Ceramide_-_ESI-MS/MS_-_Liebisch_et_al.)](https://www.lipidomicnet.org/index.php/Ceramide_-_ESI-MS/MS_-_Liebisch_et_al.)^6^

| Sample preparation | - Lipid extraction according to Bligh and Dyer^2^ - Internal standards are added prior lipid extraction - To avoid loss of lipids, extraction was performed in glassware - Samples are dissolved in methanol containing 5 mM ammonium acetate/chloroform (3/1 = v/v)  \| Material \| Material used \| Internal Standard(s) \| Internal Standard(s) added \| \| --- \| --- \| --- \| --- \| \| Cultured cells \| 100µg protein \| Cer 14:0, Cer 17:0 \| 50ng each \| \| Human plasma \| 20µl \| Cer 14:0, Cer 17:0 \| 50ng each \| |
| --- | --- | --- | --- | --- | --- | --- | --- | --- | --- | --- | --- | --- | --- |
| Instrumentation and method | Pump   - Type: binary high pressure gradient (Agilent 1100) - Mode: isocratic flow gradient - Solvent(s): Methanol containing 10 mM ammonium acetate/chloroform (3/1 = v/v) - Flow gradient:  \| Time [min] \| Flow [ml/min] \| % Solvent A \| % Solvent B \| \| --- \| --- \| --- \| --- \| \| 0 \| 0.05 \| 100 \| 0 \| \| 0.1 \| 0.03 \| 100 \| 0 \| \| 1.1 \| 0.2 \| 100 \| 0 \| \| 1.3 \| 0.05 \| 100 \| 0 \|   Autosampler   - Type: CTC Pal - Injection volume: 20µl - Wash solvent: methanol/chloroform = 1/1   Mass spectrometer   - Type: Triple quadrupole (Micromass, Quattro Ultima) - Source temperature: 250°C - Ionization mode: ESI positive - Ionization voltage: 3500 V - Collision gas: Argon - Collision gas pressure: 1.0 10^-3^ Torr - Collision energy: 30 V - recursor ion scan of m/z 264.2 - multiple reaction monitoring table of species observed frequently  \| Analyte \| Precursor [m/z] \| Precursor [m/z] \| Collision energy [eV] \| \| --- \| --- \| --- \| --- \| \| Cer 14:0 - H2O (IS) \| 492.5 \| 264.2 \| 25 V \| \| Cer 14:0 (IS) \| 510.5 \| 264.2 \| 25 V \| \| Cer 16:0 - H2O \| 520.5 \| 264.2 \| 25 V \| \| Cer 16:0 \| 538.5 \| 264.2 \| 25 V \| \| Cer 17:0 - H2O (IS) \| 534.5 \| 264.2 \| 25 V \| \| Cer 17:0 (IS) \| 552.5 \| 264.2 \| 25 V \| \| Cer 18:0 - H2O \| 548.5 \| 264.2 \| 25 V \| \| Cer 18:0 \| 566.6 \| 264.2 \| 25 V \| \| Cer 20:0 - H2O \| 576.6 \| 264.2 \| 25 V \| \| Cer 20:0 \| 594.6 \| 264.2 \| 25 V \| \| Cer 22:1 - H2O \| 602.6 \| 264.2 \| 25 V \| \| Cer 22:1 \| 620.6 \| 264.2 \| 25 V \| \| Cer 22:0 - H2O \| 604.6 \| 264.2 \| 25 V \| \| Cer 22:0 \| 622.6 \| 264.2 \| 25 V \| \| Cer 23:0 - H2O \| 618.6 \| 264.2 \| 25 V \| \| Cer 23:0 \| 636.6 \| 264.2 \| 25 V \| \| Cer 24:1 - H2O \| 630.6 \| 264.2 \| 25 V \| \| Cer 24:1 \| 648.6 \| 264.2 \| 25 V \| \| Cer 24:0 - H2O \| 632.6 \| 264.2 \| 25 V \| \| Cer 24:0 \| 650.6 \| 264.2 \| 25 V \| |
| Data analysis and quantification | Data handling   - Combine spectra above half peak height - Smooth combined spectrum (if necessary), centroid combined spectrum, pick peak intensities   Isotope correction   - Using Excel Macros correcting the peak intensities in a sequential algorithm starting from low mass species - Five isotope peaks including the monoisotopic were used - The detailed algorithm is described in the appendix of Liebisch G, et al’s study^3^   Calibration and quantification   - Calibration type: matrix calibration - addition of naturally occurring species - Both [M+H]+ and [M+H-H2O]+ are use - Species used for calibration:  \| Species \| Cultured cells \| Human plasma \| \| --- \| --- \| --- \| \| Cer 16:0 \| 0 - 35 pmol \| 0 - 50 pmol \| \| Cer 18:0 \| 0 - 35 pmol \| 0 - 50 pmol \| \| Cer 20:0 \| 0 - 35 pmol \| 0 - 50 pmol \| \| Cer 24:1 \| 0 - 35 pmol \| 0 - 50 pmol \| \| Cer 24:0 \| 0 - 35 pmol \| 0 - 50 pmol \| |
| Method validation | Precision   - CV over all: below 10% in plasma samples   Detection limit   - 0.3 pmol injected amount (in fibroblast lipid extract)   Recovery   - Recovery of Cer 6:0 above 90% |

**References**

1. Liebisch, G., Drobnik, W., Lieser, B. & Schmitz, G. High-throughput quantification of lysophosphatidylcholine by electrospray ionization tandem mass spectrometry. *Clin. Chem.* **48**, 2217–2224 (2002).

2. Bligh, E. G. & Dyer, W. J. A rapid method of total lipid extraction and purification. *Can. J. Biochem. Physiol.* **37**, 911–917 (1959).

3. Liebisch, G., Lieser, B., Rathenberg, J., Drobnik, W. & Schmitz, G. High-throughput quantification of phosphatidylcholine and sphingomyelin by electrospray ionization tandem mass spectrometry coupled with isotope correction algorithm. *Biochim. Biophys. Acta* **1686**, 108–117 (2004).

4. Zemski Berry, K. A. & Murphy, R. C. Electrospray ionization tandem mass spectrometry of glycerophosphoethanolamine plasmalogen phospholipids. *J. Am. Soc. Mass Spectrom.* **15**, 1499–1508 (2004).

5. Brügger, B., Erben, G., Sandhoff, R., Wieland, F. T. & Lehmann, W. D. Quantitative analysis of biological membrane lipids at the low picomole level by nano-electrospray ionization tandem mass spectrometry. *Proc. Natl. Acad. Sci. U. S. A.* **94**, 2339–2344 (1997).

6. Liebisch, G. *et al.* Quantitative measurement of different ceramide species from crude cellular extracts by electrospray ionization tandem mass spectrometry (ESI-MS/MS). *J. Lipid Res.* **40**, 1539–1546 (1999).
