# Supplementary material for: A multi-omics study of circulating phospholipid markers of blood pressure
Source: Sci Rep. 2022 Jan 12;12:574. doi: 10.1038/s41598-021-04446-7 (PMC8755711; doi:10.1038/s41598-021-04446-7)
Supplement: Supplementary file 2 — Supplementary Figures. [file 41598_2021_4446_MOESM2_ESM.pdf]

Supplementary Figures

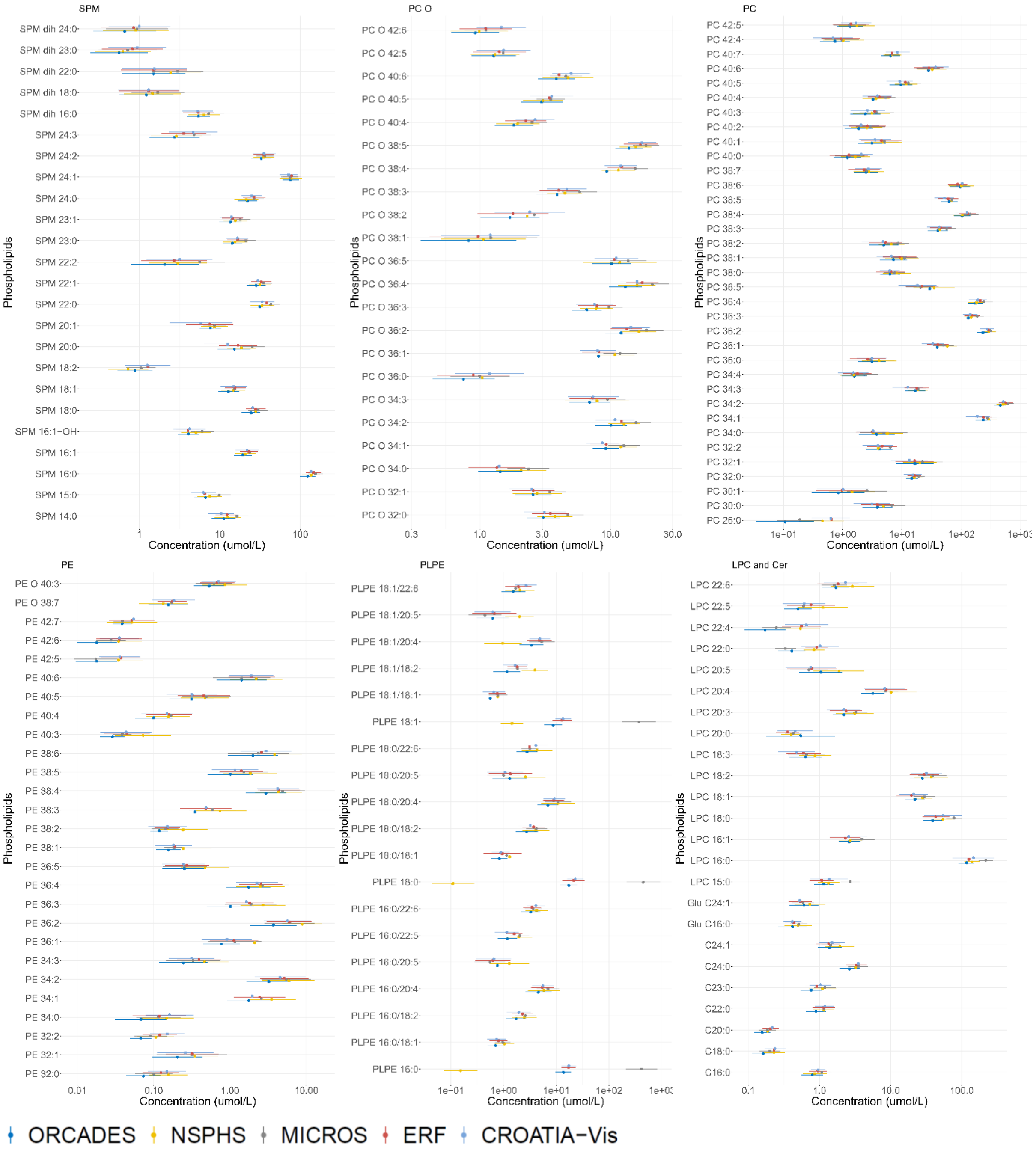

Supplementary Figure 1 Mean and standard deviation of concentration (µmol/L) of each phospholipid across cohorts. The figure is a visualization of Supplementary Table 2. It shows that all the measured phospholipids across the five cohorts are consistent except for three PLPEs (i.e., PLPE 18:1, PLPE 18:0 and PLPE 16:0) which have much higher values in CROATIA-Vis cohort than the rest four cohorts and much lower values in NSPHS than the rest four cohorts. It implies that the three phospholipids, which are not significantly associated with blood pressure, are more population stratification driven than others. We further compared the values with the publication by Trabado S, et al which studied the reference values of the phospholipids in 800 French healthy volunteers[1]. Within the overlapped phospholipids (7 LPCs, 29 PCs, 19 PC Os and 7 SPMs), we found that 89% (55/62) of the mean values in the current study are in the extreme value range reported by Trabado S, et al,

while the rest 7 (i.e., PC 38:0, PC 38:1, PC 40:1, PC 40:2, PC 42:4, LPC 16:0, LPC 18:0) are also close to the range but not found to be associated with blood pressure. However, our study was performed in five populations which are family-based and with more aging, obese and morbidity participants (Table 1), compared with the healthy and majority-young volunteers studied by Trabado S, et al. Thus, the mean values of the phospholipids in the current study are generally higher/lower than the mean values in Trabado S, et al's study based on the favourable characteristics of the specific phospholipids.

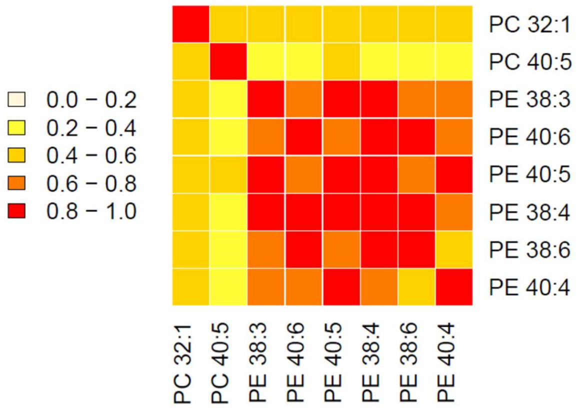

Supplementary Figure 2 Heatmap of the correlation between the phospholipids associated with blood pressure. The correlation was tested by Pearson's correlation in ERF (n=818).

References

1. Trabado, S. et al. The human plasma-metabolome: Reference values in 800 French healthy volunteers; impact of cholesterol, gender and age. PloS One 12, e0173615 (2017).
